# Supplementary material for: Rare genetic variants in genes and loci linked to dominant monogenic developmental disorders cause milder related phenotypes in the general population
Source: Am J Hum Genet. 2022 Jun 13;109(7):1308–16. doi: 10.1016/j.ajhg.2022.05.011 (PMC9300873; doi:10.1016/j.ajhg.2022.05.011)
Supplement: Document S1. Figure S1 and supplemental methods [file mmc1.pdf]

The American Journal of Human Genetics, Volume *109*

Supplemental information

Rare genetic variants in genes and loci linked to dominant monogenic developmental disorders cause milder related phenotypes in the general population

Rebecca Kingdom, Marcus Tuke, Andrew Wood, Robin N. Beaumont, Timothy M. Frayling, Michael N. Weedon, and Caroline F. Wright

Supplementary Information

Supplementary Figures

Supplementary Figure 1: Histogram showing the Variant Allele Fraction (VAF) of variants located in *DNMT3A*, dashed line indicating the average VAF of variants in other genes, with filled line showing the average VAF of variants in *DNMT3A*.

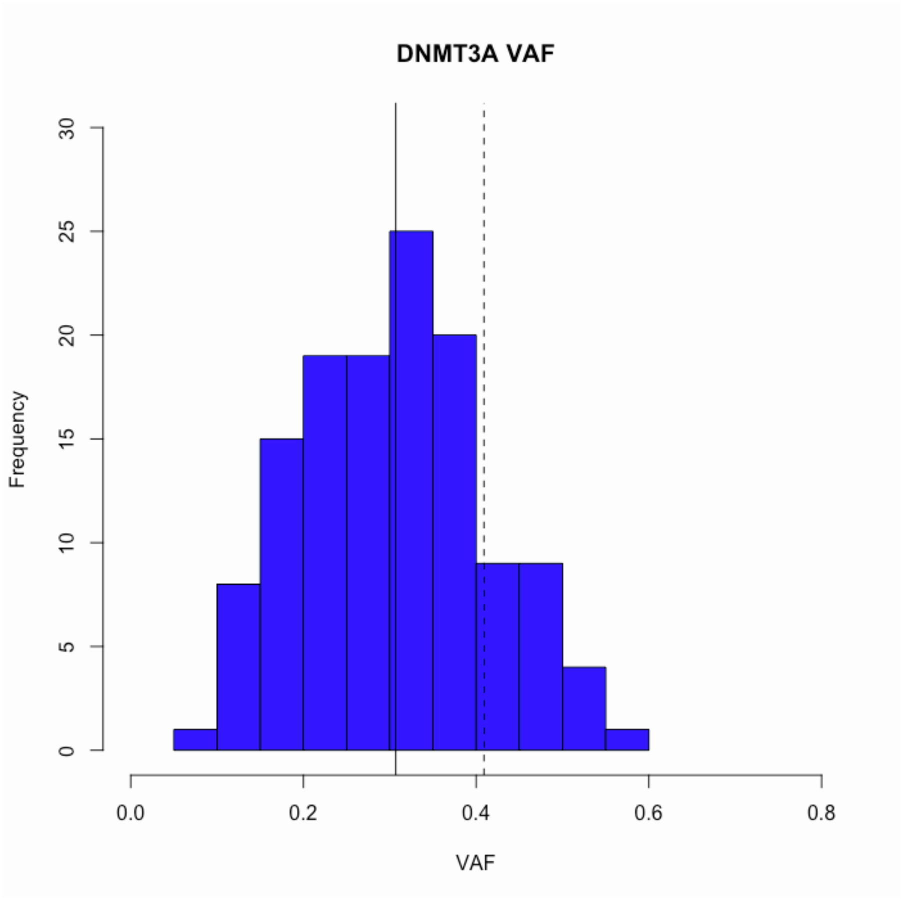

Supplementary Tables

Included in an Excel file.

Worksheet 1: Known monoallelic DDG2P genes

Worksheet 2: Copy number variants in UKB overlapping 69 known DD loci

Worksheet 3: Gene panel association tests for LoF variants across different gene subsets

Worksheet 4: Gene panel association tests for deleterious missense variants across different CADD bins

Worksheet 5: Gene panel association tests excluding individuals diagnosed with a childhood developmental disorder

Supplementary Methods

STATA code: Logistic regression

```
<import UKB BOLT phenotype file>
<import UKB exome file>
<import pcs file>
<import list of traits to test - varlist>

#varlist includes following traits: employed degree infertile epilepsy_ukbdef ChildDD_any
AdultDD_any unable_to_work

#for LoF variants:
generate variant = 1 if v2 == “lof_variant”
generate dont_use = 1 if v2 == “missense_variant”
replace variant = 0 if variant != 1
replace dont_use = 0 if dont_use != 1

file open myfile using <file_to_save_to>,
write append
set more off

foreach var of varlist {
        logistic `var' variant centre sex age_base pcs* if valid_exome =1 & dont_use != 1

file write myfile "`var'" _tab (r(table)[1,1]) _tab (r(table)[2,1]) _tab (r(table)[4,1]) _tab
(r(table)[5,1]) _tab (r(table)[6,1]) _n
}

file close myfile
```

STATA code: Linear regression

```
<import UKB BOLT phenotype file>
<import UKB exome file>
<import pcs file>
<import list of traits to test - varlist>

#varlist includes following traits: fluid_intelligence edu_yrs income reaction_time_raw_sin
pairs_test tdi age_education height mental_health numeric_memory_raw_sin
bmi_2016_raw_sin kids_fathered all_preg n_stillbirth

#for LoF variants:
generate variant = 1 if v2 == “lof_variant”
generate dont_use = 1 if v2 == “missense_variant”
replace variant = 0 if variant != 1
replace dont_use = 0 if dont_use != 1
```

```
file open myfile using <file_to_save_to>,
write append
set more off

foreach var of varlist {
	regress `var' variant centre age_base sex pcs* if valid_exome = 1 & dont_use != 1

file write myfile "`var'" _tab (r(table)[1,1]) _tab (r(table)[2,1]) _tab (r(table)[4,1]) _tab
(r(table)[5,1]) _tab (r(table)[6,1]) _n
}

file close myfile
```
