# Supplementary material for: Rare genetic variants in genes and loci linked to dominant monogenic developmental disorders cause milder related phenotypes in the general population
Source: Am J Hum Genet. 2022 Jun 13;109(7):1308–16. doi: 10.1016/j.ajhg.2022.05.011 (PMC9300873; doi:10.1016/j.ajhg.2022.05.011)
Supplement: Document S2. Article plus supplemental information [file mmc3.pdf]

Rare genetic variants in genes and loci linked to dominant monogenic developmental disorders cause milder related phenotypes in the general population

Authors

Rebecca Kingdom, Marcus Tuke, Andrew Wood, Robin N. Beaumont, Timothy M. Frayling, Michael N. Weedon, Caroline F. Wright

Correspondence

caroline.wright@exeter.ac.uk

Kingdom et al., 2022, The American Journal of Human Genetics *109*, 1308–1316
July 7, 2022 © 2022 The Author(s).
https://doi.org/10.1016/j.ajhg.2022.05.011

CellPress

ARTICLE

Rare genetic variants in genes and loci linked to dominant monogenic developmental disorders cause milder related phenotypes in the general population

Rebecca Kingdom,[1] Marcus Tuke,[1] Andrew Wood,[1] Robin N. Beaumont,[1] Timothy M. Frayling,[1] Michael N. Weedon,[1] and Caroline F. Wright[1,*]

Summary

Many rare monogenic diseases are known to be caused by deleterious variants in thousands of genes, however the same variants can also be found in people without the associated clinical phenotypes. The penetrance of these monogenic variants is generally unknown in the wider population, as they are typically identified in small clinical cohorts of affected individuals and families with highly penetrant variants. Here, we investigated the phenotypic effect of rare, potentially deleterious variants in genes and loci where similar variants are known to cause monogenic developmental disorders (DDs) in a large population cohort. We used UK Biobank to investigate phenotypes associated with rare protein-truncating and missense variants in 599 monoallelic DDG2P genes by using whole-exome-sequencing data from ~200,000 individuals and rare copy-number variants overlapping known DD loci by using SNP-array data from ~500,000 individuals. We found that individuals with these likely deleterious variants had a mild DD-related phenotype, including lower fluid intelligence, slower reaction times, lower numeric memory scores, and longer pairs matching times compared to the rest of the UK Biobank cohort. They were also shorter, had a higher BMI, and had significant socioeconomic disadvantages: they were less likely to be employed or be able to work and had a lower income and higher deprivation index. Our findings suggest that many genes routinely tested within pediatric genetics have deleterious variants with intermediate penetrance that may cause lifelong sub-clinical phenotypes in the general adult population.

Introduction

Deleterious variants in thousands of genes have been shown to cause rare, monogenic diseases.[1] However, not all individuals with these variants share the same clinical phenotypes; some don't appear to be affected at all, whereas others are very severely affected.[2] Monogenic variants can display different effects in different individuals.[3] The range of phenotypes caused by deleterious variants in the same gene can be explained by pleiotropy, incomplete penetrance, and variable expressivity.[4] Penetrance (i.e., whether an individual with a disease-causing genotype displays the corresponding clinical phenotype) is generally binary; either a variant is penetrant and causes the clinical phenotype associated with that genotype or it is not.[2,5] In contrast, variable expressivity (i.e., the range of phenotypes that can be observed in affected individuals) is generally continuous, e.g., from mild to severe.[6] Although incomplete penetrance and variable expressivity are distinct concepts, in practice they can be hard to separate, especially when considering continuous phenotypes in populations.

As most monogenic disease-causing variants have been identified through small clinical cohorts, including families with multiple affected individuals, penetrance of these variants is often overestimated. Investigating the effect of these variants in the general population is therefore important to give a more accurate view of the penetrance in clinically unselected individuals and families. It has been suggested that many of the primary symptoms of rare disease are actually extremes of normally distributed phenotypes in the general population.[1,7] Large, well genotyped population cohorts give us the ability to investigate the spectrum of phenotypes of individuals with variants in genes known to cause monogenic disease. Phenotypic heterogeneity and variability are a major concern for rare Mendelian disorders, where they can lead to incorrect or delayed diagnoses.[8,9]

Many severe developmental disorders (DDs) manifest from birth or early childhood and are caused by rare damaging variants in around 2,000 genes and loci.[10] Pathogenic variants in these genes have been identified primarily through phenotype-led clinical studies of affected individuals and families.[4] Due to extensive genetic and phenotypic heterogeneity, large multigene panels are increasingly being used for diagnostic testing, often through panel-based virtual analysis of whole-exome-sequence or whole-genome-sequence data. However, little is known about what effect, if any, deleterious variants in these genes have on adults in the general population or their lifelong implications. In this study, using genetic and phenotypic data from UK Biobank (UKB),[11] we investigated whether adults with rare deleterious variants in genes and loci linked to dominant monogenic DD have any developmentally relevant phenotypes.

[1]Institute of Biomedical and Clinical Science, University of Exeter College of Medicine and Health, RILD Building, Barrack Road, Exeter EX2 5DW, UK
*Correspondence: caroline.wright@exeter.ac.uk
https://doi.org/10.1016/j.ajhg.2022.05.011.
© 2022 The Author(s). This is an open access article under the CC BY license (http://creativecommons.org/licenses/by/4.0/).

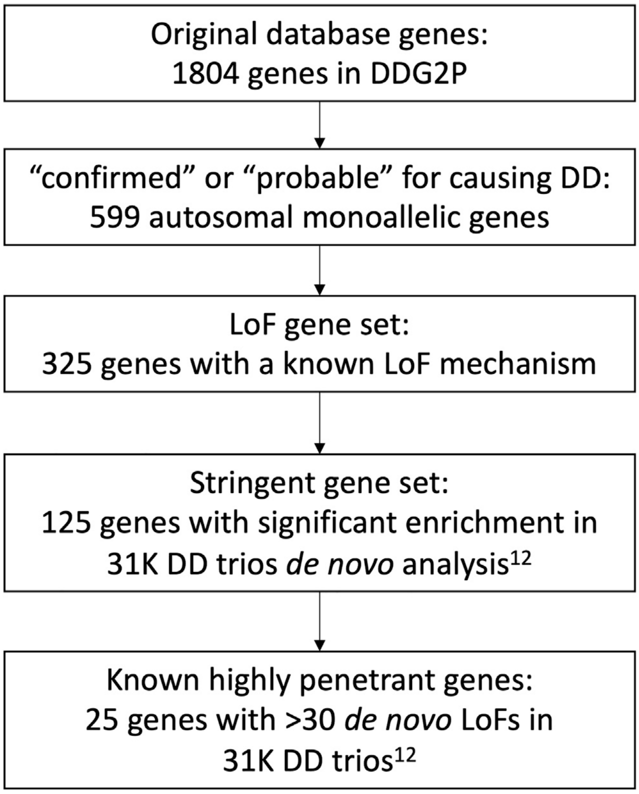

Figure 1. Flow diagram outlining selection process for genes in each subset that were used for analysis
DDG2P, Developmental Disorders Genotype-to-Phenotype database; DD, developmental disorder; LoF, loss-of-function variants; 31K DD trios, 31,058 parent-offspring families with developmental disorders.[12]

Material and methods

UK biobank cohort

UKB is a population-based cohort from the UK with deep phenotyping data and genetic data for around 500,000 individuals aged 40–70 years at recruitment. Individuals provided a variety of information via self-report questionnaires, cognitive and anthropometric measurements, and Hospital Episode Statistics (HES) including ICD9 and ICD10 codes. Genotypes for single-nucleotide polymorphisms (SNPs) were generated with the Affymetrix Axiom UK Biobank array (~450,000 individuals) and the UK BiLEVE array (~50,000 individuals). This dataset underwent extensive central quality control (http://biobank.ctsu.ox.ac.uk). A subset of ~200,000 individuals also underwent whole-exome sequencing (WES) with the IDT xGen Exome Research Panel v1.0; this dataset was made available for research in October 2020. Detailed sequencing and variant detection methodology for UKB is available at https://biobank.ctsu.ox.ac.uk/showcase/label.cgi?id=170. The UKB resource was approved by the UK Biobank Research Ethics Committee and all participants provided written informed consent to participate.

Gene selection

We used the clinically curated Developmental Disorders Gene2Phenotype Database (DDG2P; https://www.ebi.ac.uk/gene2phenotype/) to select genes where rare variants are known to cause monogenic DD. The database (accessed on 27 November 2020) was constructed from published literature and provides information relating to genes, variants, and phenotypes associated with DDs, including mode of inheritance and mechanism of pathogenicity.[10] We initially included all genes that had been annotated as monoallelic (i.e., autosomal dominant) with an evidence level of "confirmed" or "probable" (n = 599). Further subsets of these genes were selected for sensitivity analyses, including a panel of 325 genes where variants are known to cause DD through a loss-of-function (LoF) haploinsufficiency mechanism; a more stringent panel of 125 of these genes that were significantly enriched for damaging *de novo* LoF mutations in a recent analysis of 31,058 DD probands;[12] and a small panel of 25 genes where deleterious variants cause clinically well-established syndromes, with >30 likely pathogenic *de novo* LoF mutations in the same study[12] (see Figure 1 and Table S1).

Variant selection

To investigate the penetrance of likely deleterious single-nucleotide variants (SNVs) and insertions/deletions (indels) in genes where rare variants are known to cause autosomal dominant DD, we used WES data from 200,632 individuals in UKB to identify individuals with a rare SNVs and/or indels in any of these genes. For most of our analyses, rare was defined as any variant that occurred in 5 or fewer individuals in the UKB cohort; we also investigated the effect of changing this threshold to n = 1, n = 10, n = 50, and n = 100 individuals. We included variants that had individual and variant missingness < 10%, minimum read depth of 7 for SNVs and 10 for indels, and at least one sample per site passed the allele balance threshold > 15% for SNVs and 20% for indels. We selected three functional classes of variant in canonical transcripts based on annotation in genome build GRCh38 by the Ensembl Variant Effect Predictor.[13]

(1) Likely deleterious LoF variants: we defined an LoF variant as one that is predicted to cause a premature stop, a frameshift, or abolish a canonical splice site; only those variants deemed to be high confidence by the Loss-Of-Function Transcript Effect Estimator (LOFTEE) were retained (https://github.com/konradjk/loftee).
(2) Likely deleterious missense variants: missense variants with a REVEL score > 0.7.[14] A further set of likely deleterious missense variants were identified via CADD,[15] with scores of >20, >25, and >30.
(3) Likely benign synonymous variants.

Individuals with variants in group 1 were excluded from groups 2 and 3; individuals with variants in group 2 were excluded from group 3. Following variant selection, one gene (*DNMT3A*) was removed from further analysis as the variants in this gene—which is known to be strongly linked to blood cancer[16]—had a significantly lower allele balance, suggesting substantial somatic mosaicism (see Figure S1). Other genes linked to blood cancer, such as *ASXL1* and *TET3*, were examined but showed no difference in allele balance compared to the remainder of the LoF variants identified. LoF variants in the most stringent 25 gene subset were visually confirmed with the Integrative Genomics Viewer (IGV).

To investigate the penetrance of multigenic copy-number variants (CNVs) overlapping known DD loci, we used SNP-array data from 488,377 genotyped individuals in UKB and

PennCNV[17] (version 1.0.4) to detect multigenic CNVs overlapping 69 published CNVs strongly associated with developmental delay.[18,19] Log R ratio (LRR) and B-allele frequency (BAF) values for 805,426 genome-wide SNP probe sets were provided by UKB, and we used an in-house script to convert these data to PennCNV input signal files. The PennCNV hidden Markov model (HMM) transition matrix was trained with 250 random UKB samples with PennCNV-train. Population Frequency B Allele reference data (PFB) were generated via 1,000 random UKB samples. We then used PennCNV-test to detect regions in a duplication or deletion state in LRR/BAF HMM with the generated PFB and transition matrix. An individual was classified as having a multigenic DD deletion or duplication if the region detected with PennCNV reciprocally intersected the published region by at least 50%. We plotted LRR/BAF data for each call in each of these regions, and carried out visual inspection of each event, and false positives and single gene CNVs were excluded. A list of included CNVs is provided in Table S2.

Statistical analysis

We performed both individual gene and gene panel burden tests across our different gene subsets. We grouped individuals into one of five groups depending upon the type of variant they carried (LoF, missense, or synonymous variant in one or more monoallelic DDG2P gene or deletion or duplication overlapping published DD multigenic CNVs). Association tests were limited to individuals in UKB with genetically defined European ancestry that were unrelated up to third-degree relationship (184,142 with WES data; 380,029 with SNP-array data) and were controlled for age, sex, recruitment center, and 40 principal components. Variant burden association tests in gene panels and multigenic CNVs were performed with STATA (version 16.0) with linear regression for continuous phenotypes and logistic regression for the binary phenotypes. Associations were tested between each group of individuals and other individuals in the UKB cohort without any of the classes of rare variation defined above. Information from HES codes, self-report questionnaires, and cognitive tests taken at recruitment was used for the phenotypic information. Associations were tested for 20 UKB phenotypes selected on the basis of their likely relevance to DDs, including the following.

- Medical: epilepsy (self-reported or ICD10 codes G40); ever reported a mental health issue (self-reported through questionnaire); diagnosed with "child DD" (including intellectual disability [ICD10 codes F70-73], epilepsy [G40], developmental disorders [F80-84], and congenital malformations [Q0-99]); or diagnosed "adult DD" (including schizophrenia, [self-reported or ICD10 codes F20-29] and bipolar disorder [self-reported or ICD10 codes F30-F39]).
- Reproductive: number of pregnancies, number of stillbirths, number of children fathered.
- Physical: height, body mass index (BMI) (inverse normalized).
- Cognitive: fluid intelligence (field ID: 20,016), reaction time (inverse normalized, field ID: 20023), pairs matching score (field ID: 20131), numeric memory score (inverse normalized, field ID: 20240), age left education, number of years in education, has a degree.
- Socioeconomic: in employment, unable to work (both field ID: 6142), income (field ID: 738), Townsend deprivation index (TDI) (field ID: 189).

Results

Many individuals in UKB carry rare, deleterious variants in genes where similar variants are known to cause monogenic autosomal dominant DD

Although variants in each gene individually account for extremely rare forms of DD, together they account for a large portion of DD diagnoses and have a surprisingly high burden of rare deleterious variants in UKB. In 184,477 unrelated European individuals with WES data in UKB and across 599 monoallelic DDG2P genes, 9,103 individuals carry a rare ($n \leq 5$) LoF variant, 25,288 individuals carry a rare missense variant with REVEL > 0.7, and 79,959 individuals carry a rare synonymous variant. As the gene panel becomes smaller and more stringent, the burden of rare deleterious variants decreases; for example, 3,602, 1,327, and 167 individuals in UKB carry rare LoF variants in smaller more stringent subsets of 325, 125, and 25 DDG2P genes, respectively (Figure 1). In 450,274 individuals with SNP-array data in UKB and across 69 known DD loci, 4,922 individuals carry large deletions and 7,054 individuals carry large duplications.

Individuals in UKB with rare, deleterious variants in loci where similar variants are known to cause monogenic DD display DD-related phenotypes

We performed gene panel (including 599 monoallelic DDG2P genes) and multigenic copy-number (including 53 deletions/duplications syndromes) burden tests for 20 traits in UKB selected to be of relevance (in adults) to developmental phenotypes. Bonferroni-corrected significant associations were found across most phenotypes in individuals carrying likely damaging variants compared with the rest of the UKB cohort (Table 1, Figure 2, and Figure 3). Individuals carrying these variants generally had lower cognitive performance than the rest of the cohort, with reduced fluid intelligence (LoF group beta: -1.059), slower reaction times (LoF group beta: $+0.043$), lower numeric memory scores (LoF group beta: -0.068), and longer pairs matching times (LoF group beta: $+0.122$). They also completed fewer years in education, left education at an earlier age, and were less likely to have a degree. Medically, individuals were more likely to have reported a mental health issue or been diagnosed with either a childhood DD (including mild-severe intellectual disability, epilepsy, autism, ADHD, and congenital malformations) or adult DD (including schizophrenia and bipolar disorder). Individuals were also more likely to be shorter, have a higher BMI, and have had fewer children (though the latter association was only significant in men). Individuals also had significant socioeconomic disadvantages: they were less likely to be employed or be able to work and had a lower income and a higher TDI. Across all phenotypes tested, we observed a trend corresponding to the likely deleteriousness of the variants; the largest effect was generally observed in the group of individuals with multigenic

Table 1. Gene panel association tests results

Dataset	Deletions overlapping 69 DD loci		Duplications overlapping 69 DD loci		LoF variants in 599 DDG2P genes		Missense variants (REVEL > 0.7) in 599 DDG2P genes		Synonymous variants in 599 DDG2P genes	
**Binary traits**	**OR**	**p value**	**OR**	**p value**	**OR**	**p value**	**OR**	**p value**	**OR**	**p value**
In employment	0.728	3.356E−10	0.814	7.580E−7	0.907	5.778E−4	0.988	0.500	1.012	0.323
Have a degree	0.624	2.052E−28	0.748	6.684E−17	0.833	6.134E−15	0.925	1.368E−7	1.028	6.115E−3
Have an epilepsy diagnosis	1.689	2.179E−3	1.292	0.113	1.394	0.003	1.068	0.403	0.917	0.131
Diagnosed with child DD	1.588	1.827E−4	1.279	0.030	1.316	5.056E−4	1.123	0.031	1.018	0.645
Diagnosed with adult DD	1.502	1.359E−6	1.395	4.027E−6	1.158	7.061E−3	1.062	0.092	1.003	0.914
Is unable to work	1.921	1.093E−16	1.554	6.663E−10	1.344	8.573E−8	1.134	8.459E−4	0.977	0.403
**Continuous traits**	**Beta**	**p value**	**Beta**	**p value**	**Beta**	**p value**	**Beta**	**p value**	**Beta**	**p value**
Fluid intelligence	−0.592	3.834E−20	−0.347	2.534E−11	−0.159	1.152E−6	−0.089	1.207E−5	0.002	0.865
Number of years in education	−1.139	7.878E−30	−0.755	1.496E−19	−0.391	4.589E−12	−0.189	1.323E−7	0.064	0.009
Income	−0.346	1.850E−45	−0.217	1.042E−26	−0.127	1.599E−20	−0.058	2.675E−11	0.012	0.040
Reaction time	0.199	1.086E−25	0.079	6.277E−7	0.043	8.179E−5	0.013	0.060	−0.005	0.290
Pairs test score	0.285	1.345E−5	0.315	7.174E−9	0.122	9.928E−4	0.055	0.019	−0.022	0.172
Townsend deprivation index	0.527	8.628E−19	0.485	9.962E−23	0.279	5.596E−17	0.090	1.855E−5	0.020	0.160
Age left education	−0.214	2.158E−5	−0.218	4.345E−7	−0.110	2.892E−4	−0.044	0.025	0.003	0.806
Height	−1.608	1.474E−36	−0.613	7.254E−9	−0.449	4.809E−10	−0.251	3.725E−8	0.044	0.164
Reported a mental health issue	0.071	1.629E−3	0.023	0.222	0.041	1.047E−3	0.015	0.053	−0.001	0.848
Numeric memory score	−0.188	1.765E−6	−0.054	0.096	−0.068	1.032E−3	−0.025	0.053	−0.002	0.813
BMI	0.157	3.164E−15	0.112	1.766E−11	0.032	4.611E−3	0.016	0.024	−0.003	0.608
Number of children fathered	−0.216	1.048E−9	−0.100	6.985E−4	−0.069	1.135E−3	−0.018	0.168	−0.011	0.210
Number of pregnancies	−0.041	0.358	−0.039	0.292	−0.043	0.076	−0.024	0.120	0.007	0.499
Number of stillbirths	0.005	0.381	0.009	0.066	0.004	0.245	0.004	0.039	0.001	0.430

DD, developmental disorder; LoF, loss of function; OR, odds ratio; BMI, body mass index.
Twenty relevant phenotypes were tested in individuals in UK Biobank carrying deletions or duplications overlapping 69 known DD-associated loci or rare ($n \leq 5$) LoF, missense (REVEL > 0.7), or synonymous variants in any of 599 known monoallelic DDG2P genes.

deletions, followed by multigenic duplications, then LoF variants, and finally missense variants in one (or more) DDG2P genes. These trends were robust to the use of different CADD thresholds for selecting of missense variants (see Table S4) and to removal of individuals with a diagnosed childhood developmental disorder ("child DD," as defined in material and methods, n = 3,132; see Table S5). In contrast, individuals with only rare synonymous variants in these genes showed no statistically significant difference in any phenotype compared to the remainder of the cohort, as expected for likely benign variants, suggesting that most of the confounding caused by population sub-structure was appropriately controlled.

Potentially damaging LoF variants were found even in genes associated with clinically well-established developmental syndromes

We repeated our association analysis with smaller, more stringent, subsets of 325, 125, and 25 DDG2P genes (Figure 1). Interestingly, even within the most stringent subset of 25 genes where rare variants are thought to cause highly penetrant severe forms of DD via haploinsufficiency, with >30 *de novo* LoF mutations identified in 31,058 DD probands,[12] we were able to identify 167 individuals in UKB who had a high confidence LoF variant in one of these genes. We observed similar trends to the full 599 gene panel for LoF variants in smaller subsets of genes in which variants cause DD by haploinsufficiency: the group overall exhibited mild DD-related phenotypes, although the results were less significant because of the smaller number of individuals carrying likely LoF variants (Table S3). Nonetheless, a Bonferroni-corrected significant result was seen across all gene subsets for shorter stature, reduced chance of having a degree, and increased TDI; lower fluid intelligence, lower income, higher BMI, and an increased chance of being diagnosed with a child DD also remained nominally significant even in the 25 gene subset. We also performed single-gene burden testing but were under-powered to find any significant associations for most genes as a result of the small number of individuals and likely mild phenotypic effects in UKB. Interestingly, despite previously reaching genome-wide

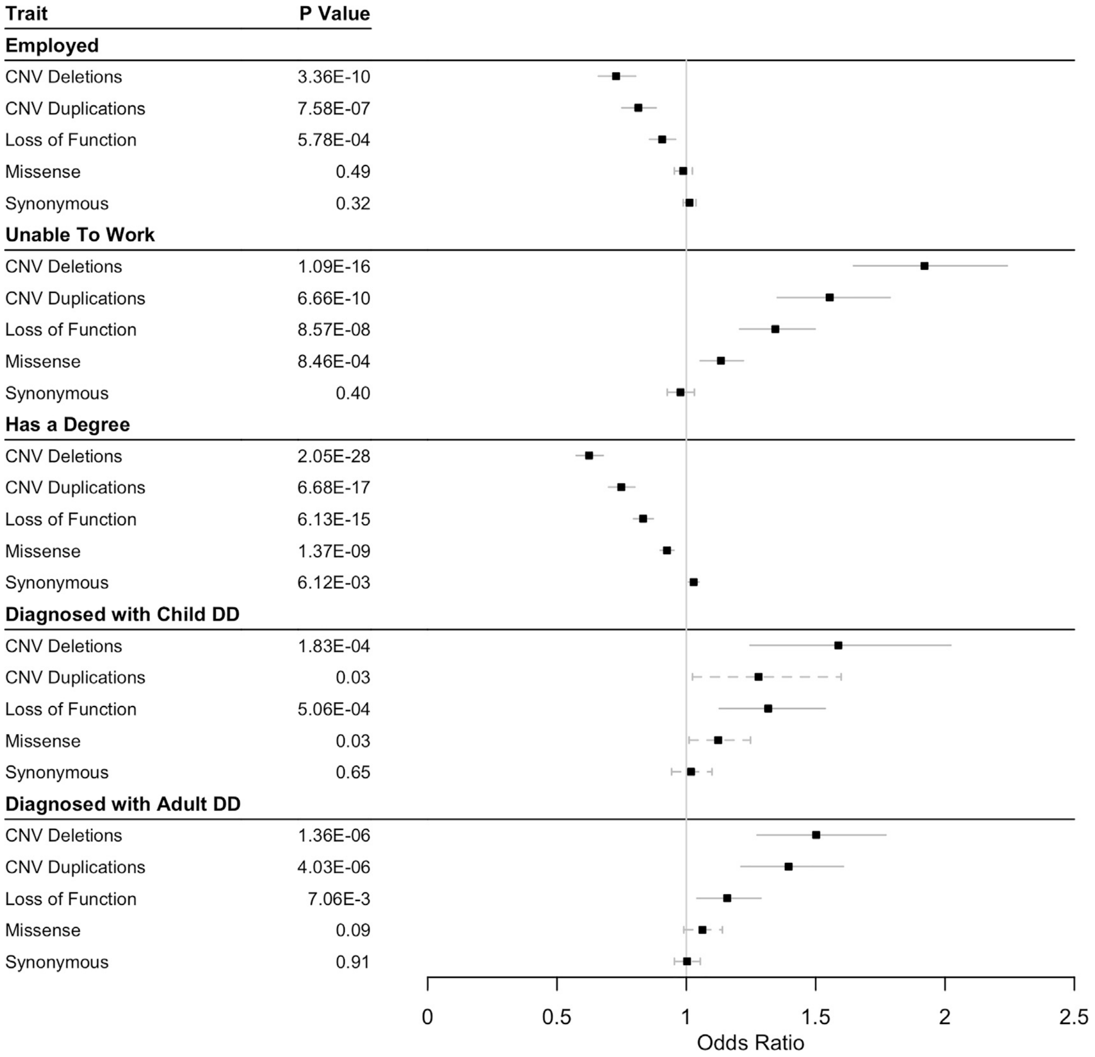

Figure 2. Summary of gene panel association tests across binary traits for carriers of likely deleterious variants
Associations are shown for individuals carrying deletions or duplications overlapping 69 known DD loci or rare ($n \leq 5$) LoF, missense (REVEL > 0.7), or synonymous variants in any of 599 known monoallelic DDG2P genes compared with the remaining unrelated White Europeans in UKB. 95% confidence intervals shown; unbroken lines, below Bonferroni-corrected p value; dashed lines, above Bonferroni-corrected p value.

significance for enrichment of damaging *de novo* mutations, *MIB1* had the largest number of individuals carrying likely LoF variants in UKB (n = 260), more than the 25 most stringent genes combined, but showed no associations with any DD-related phenotypes. The gene also has almost double the number of LoF variants observed versus expected in gnomAD (https://gnomad.broadinstitute.org/gene/MIB1), and thus appears to be remarkably unconstrained.

Rarer LoF variants have a larger phenotypic effect than more common LoF variants

We investigated the effect of allele count (AC) on the phenotypic effect of LoF variants in our largest gene panel (599 monoallelic DDG2P genes). Specifically, we performed association tests with 16 DD-related traits that were significant in the previous analysis for groups of individuals with rare LoF variants in these genes that were present in just a single individual in UKB, compared with variants seen 5, 10, 50, or 100 or fewer times (Figure 4). The group of individuals who had the rarest variants (AC = 1) had the largest phenotypic effect change compared to the rest of the cohort, although the results were generally not significant as a result of low numbers. However, across the phenotypes tested, both the effect size and the p value decreased as the AC increased, suggesting either that more common variants have a milder effect on phenotype or that more common variants are benign and are simply diluting the effect of rare pathogenic variants. No difference was observed between the effect of LoF variants in the first and second half of genes. In addition, 295 individuals had LoF variants that were previously classified as "likely pathogenic" or "pathogenic" in ClinVar, but no significant difference was detectable in their phenotypes compared with the remainder of the group who also had LoF variants.

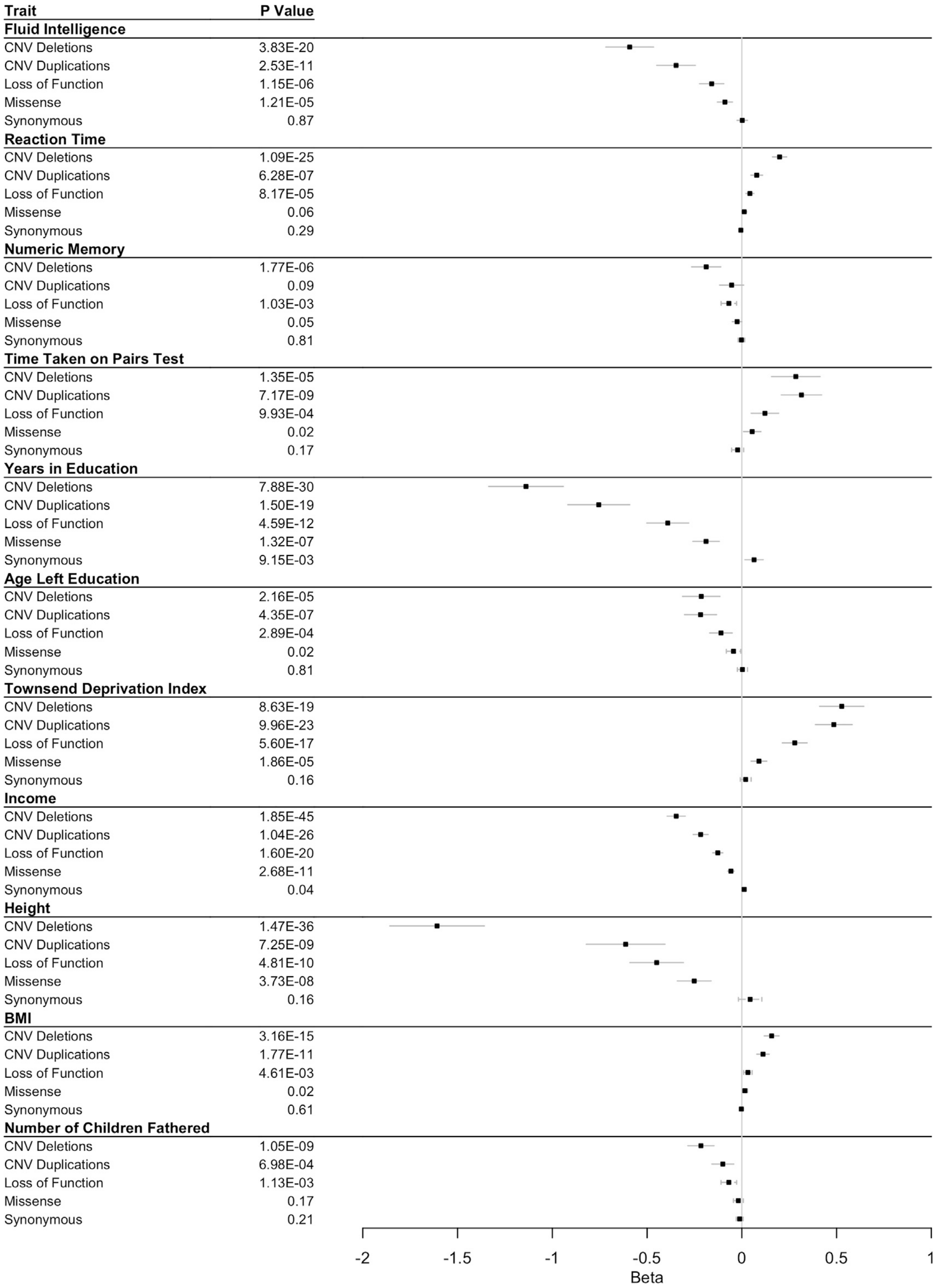

Figure 3. Summary of gene panel association tests across continuous traits for carriers of likely deleterious variants
Associations are shown for individuals carrying deletions or duplications overlapping 69 known DD-associated loci or rare ($n \leq 5$) LoF, missense (REVEL > 0.7), or synonymous variants in any of 599 known monoallelic DDG2P genes compared with the remaining unrelated White Europeans in UKB. 95% confidence intervals shown; unbroken lines, below Bonferroni-corrected p value; dashed lines, above Bonferroni-corrected p-value.

Discussion

We have shown that rare, potentially damaging variants in genes and loci linked to dominant monogenic DD are present in adults in UK Biobank and result in a mild developmental phenotype. Individuals carrying these variants have notably reduced cognitive abilities and a lower socioeconomic status. Gene panel association tests suggest a

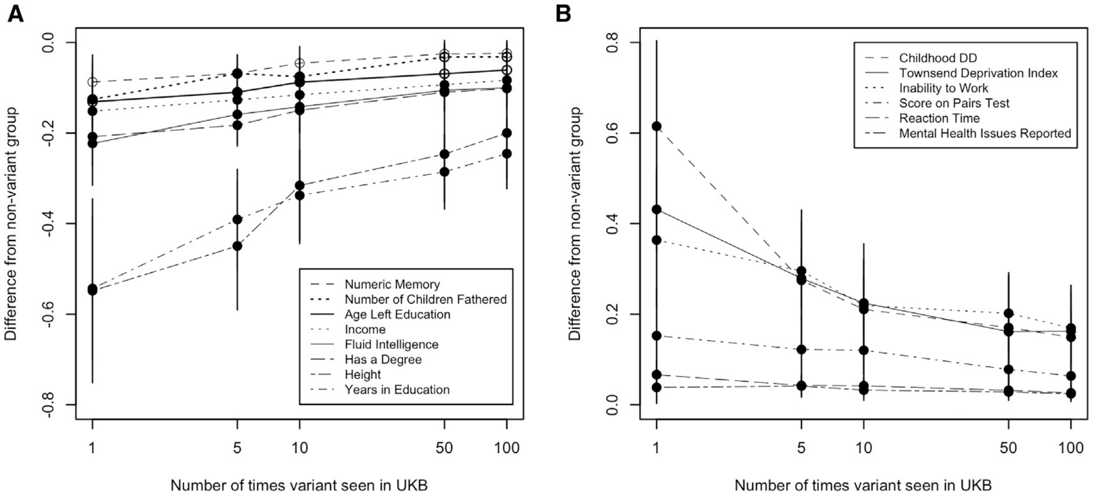

Figure 4. Change in phenotype associations for individuals with an LoF variant in 599 known monoallelic DDG2P genes versus different allele counts
Associations are grouped by whether the effect of AC = 1 LoF variants either (A) decreases or (B) increases the phenotype. 95% confidence intervals shown.

strong and consistent trend for increasing phenotypic effects with rarer and more damaging variants. Although it is impossible to disentangle incomplete penetrance and variable expressivity in a population study, our findings are consistent with similar studies in clinically unselected population cohorts[4,20–24] showing reduced penetrance of rare damaging variants in genes where similar variants cause rare monogenic forms of DD. Moreover, our results are robust to removal of individuals diagnosed with a childhood developmental disorder, suggesting that fully penetrant individuals are not driving the signal.

We note that the variants identified in UKB are not necessarily the same ones that have been identified previously in clinical cases, and indeed very few of those we identified had previously been annotated in ClinVar.[25] We also note that our dataset most likely includes some predicted-LoF variants that do not actually result in a loss of function (either because of technical false positives or biological rescue through translation re-initiation, alternative splicing, etc.). Nonetheless, these issues are common to any clinical or research scenario where variants are prioritized from WES data, and our findings were robust when limited to likely LoF variants in a subset of 325 genes in which rare variants cause DD via a haploinsufficiency mechanism. The fact that our findings are robust to smaller, more stringent subsets of genes also suggests that the low effect sizes cannot simply be explained by a subset of variants in low penetrance (or non-causal) genes. Furthermore, rare predicted-LoF variants were found in individuals in genes in which similar variants are thought to be fully or nearly fully penetrant causes of very well-established developmental syndromes, but without the full clinical phenotype that would be expected, suggesting that there is a range of penetrance and expressivity in the general population.

Despite the large size of UKB, we were limited by the number of individuals of European ancestry carrying rare damaging variants in these genes, which meant some of our analyses were under-powered to show a significant effect. We were also limited by the clinical and phenotypic data available on these individuals, all of whom were over 40 years of age at recruitment; evaluation and diagnosis of DD was much less routine when these individuals were children and is less likely to be recorded in the HES codes of older adults. Nonetheless, when found in an appropriate clinical pediatric setting, rare damaging variants in these genes are widely considered diagnostic for DD and thus they might not be expected to be present in a population cohort. Our results suggest that, although the penetrance of variants across these genes is lower than would be expected from previous clinical studies, they do still exert a phenotypic effect on adults in the general population who are nonetheless healthy enough, and have sufficient capacity, to volunteer to participate in a biobank.

Variants that cause monogenic DD have historically been identified almost exclusively through clinical cohorts of affected children and families, and their effect on adults in the general population has not previously been evaluated. While clinical studies may overestimate the penetrance of such rare variants, population cohorts like UKB are likely to underestimate the penetrance as a result of ascertainment bias toward healthy individuals.[26] The penetrance and expressivity of variants in these genes could be affected by a number of different modifiers, including genetic variants in other genes, regulatory variants affecting gene expression, somatic mosaicism, and accumulated environmental factors.[5] The latter is particularly relevant when considering the effect of damaging variants in DDG2P genes on adults. It is interesting to note that, unlike most traits, the heritability of intelligence (i.e., general cognitive ability) increases dramatically with age,[27] suggesting a major role for gene-environment

interactions as individuals become better able to select, modify, and optimize their environment. Further research is needed into the penetrance of rare, damaging variants in the general population using larger datasets, which may allow modifiers to be investigated to help explain why some individuals are more severely affected by particular genetic conditions than others.

Data and code availability

Original source data from UK Biobank is available on application from https://www.ukbiobank.ac.uk/. STATA regression analysis code is provided in supplemental information.

Supplemental information

Supplemental information can be found online at https://doi.org/10.1016/j.ajhg.2022.05.011.

Acknowledgments

This research has been conducted with the UK Biobank Resource under application number 49847. The authors would like to acknowledge funding from the University of Exeter and the MRC (MR/T00200X/1) and the use of the University of Exeter High-Performance Computing (HPC) facility in carrying out this work.

Declaration of interests

The authors declare no competing interests.

Received: December 15, 2021
Accepted: May 19, 2022
Published: June 13, 2022

References

1. Niemi, M.E.K., Martin, H.C., Rice, D.L., Gallone, G., Gordon, S., Kelemen, M., McAloney, K., McRae, J., Radford, E.J., Yu, S., et al. (2018). Common genetic variants contribute to risk of rare severe neurodevelopmental disorders. Nature *562*, 268–271. https://doi.org/10.1038/s41586-018-0566-4.
2. Gruber, C., and Bogunovic, D. (2020). Incomplete penetrance in primary immunodeficiency: a skeleton in the closet. Hum. Genet. *139*, 745–757. https://doi.org/10.1007/s00439-020-02131-9.
3. Oetjens, M.T., Kelly, M.A., Sturm, A.C., Martin, C.L., and Ledbetter, D.H. (2019). Quantifying the polygenic contribution to variable expressivity in eleven rare genetic disorders. Nat. Commun. *10*, 4897. https://doi.org/10.1038/s41467-019-12869-0.
4. Wright, C.F., West, B., Tuke, M., Jones, S.E., Patel, K., Laver, T.W., Beaumont, R.N., Tyrrell, J., Wood, A.R., Frayling, T.M., et al. (2019). Assessing the pathogenicity, penetrance, and expressivity of putative disease-causing variants in a population setting. Am. J. Hum. Genet. *104*, 275–286. https://doi.org/10.1016/j.ajhg.2018.12.015.
5. Cooper, D.N., Krawczak, M., Polychronakos, C., Tyler-Smith, C., and Kehrer-Sawatzki, H. (2013). Where genotype is not predictive of phenotype: towards an understanding of the molecular basis of reduced penetrance in human inherited disease. Hum. Genet. *132*, 1077–1130. https://doi.org/10.1007/s00439-013-1331-2.
6. Shawky, R.M. (2014). Reduced penetrance in human inherited disease. J. Med. Hum. Genet. *15*, 103–111. https://doi.org/10.1016/j.ejmhg.2014.01.003.
7. Castel, S.E., Cervera, A., Mohammadi, P., Aguet, F., Reverter, F., Wolman, A., Guigo, R., Iossifov, I., Vasileva, A., and Lappalainen, T. (2018). Modified penetrance of coding variants by cis-regulatory variation contributes to disease risk. Nat. Genet. *50*, 1327–1334. https://doi.org/10.1038/s41588-018-0192-y.
8. Rahit, K.M.T.H., and Tarailo-Graovac, M. (2020). Genetic modifiers and rare mendelian disease. Genes *11*, 239. https://doi.org/10.3390/genes11030239.
9. Maya, I., Sukenik-Halevy, R., Basel-Salmon, L., and Sagi-Dain, L. (2020). Ten points to consider when providing genetic counseling for variants of incomplete penetrance and variable expressivity detected in a prenatal setting. Acta Obstet. Gynecol. Scand. *99*, 1427–1429. https://doi.org/10.1111/aogs.13963.
10. Wright, C.F., Fitzgerald, T.W., Jones, W.D., Clayton, S., McRae, J.F., van Kogelenberg, M., King, D.A., Ambridge, K., Barrett, D.M., Bayzetinova, T., et al. (2015). Genetic diagnosis of developmental disorders in the DDD study: a scalable analysis of genome-wide research data. Lancet Lond. Engl. *385*, 1305–1314. https://doi.org/10.1016/s0140-6736(14)61705-0.
11. Bycroft, C., Freeman, C., Petkova, D., Band, G., Elliott, L.T., Sharp, K., Motyer, A., Vukcevic, D., Delaneau, O., O'Connell, J., et al. (2018). The UK Biobank resource with deep phenotyping and genomic data. Nature *562*, 203–209. https://doi.org/10.1038/s41586-018-0579-z.
12. Kaplanis, J., Samocha, K.E., Wiel, L., Zhang, Z., Arvai, K.J., Eberhardt, R.Y., Gallone, G., Lelieveld, S.H., Martin, H.C., McRae, J.F., et al. (2020). Evidence for 28 genetic disorders discovered by combining healthcare and research data. Nature *586*, 757–762. https://doi.org/10.1038/s41586-020-2832-5.
13. Thormann, A., Halachev, M., McLaren, W., Moore, D.J., Svinti, V., Campbell, A., Kerr, S.M., Tischkowitz, M., Hunt, S.E., Dunlop, M.G., et al. (2019). Flexible and scalable diagnostic filtering of genomic variants using G2P with Ensembl VEP. Nat. Commun. *10*, 2373. https://doi.org/10.1038/s41467-019-10016-3.
14. Ioannidis, N.M., Rothstein, J.H., Pejaver, V., Middha, S., McDonnell, S.K., Baheti, S., Musolf, A., Li, Q., Holzinger, E., Karyadi, D., et al. (2016). REVEL: an ensemble method for predicting the pathogenicity of rare missense variants. Am. J. Hum. Genet. *99*, 877–885. https://doi.org/10.1016/j.ajhg.2016.08.016.
15. Rentzsch, P., Witten, D., Cooper, G.M., Shendure, J., and Kircher, M. (2019). CADD: predicting the deleteriousness of variants throughout the human genome. Nucleic Acids Res. *47*, D886–D894. https://doi.org/10.1093/nar/gky1016.
16. Qi, H., Dong, C., Chung, W.K., Wang, K., and Shen, Y. (2016). Deep genetic connection between cancer and developmental disorders. Hum. Mutat. *37*, 1042–1050. https://doi.org/10.1002/humu.23040.
17. Wang, K., Li, M., Hadley, D., Liu, R., Glessner, J., Grant, S.F.A., Hakonarson, H., and Bucan, M. (2007). PennCNV: an integrated hidden Markov model designed for high-resolution copy number variation detection in whole-genome SNP genotyping data. Genome Res. *17*, 1665–1674. https://doi.org/10.1101/gr.6861907.
18. Cooper, G.M., Coe, B.P., Girirajan, S., Rosenfeld, J.A., Vu, T.H., Baker, C., Williams, C., Stalker, H., Hamid, R., Hannig, V., et al. (2011). A copy number variation morbidity map of developmental delay. Nat. Genet. *43*, 838–846. https://doi.org/10.1038/ng.909.

19. Coe, B.P., Witherspoon, K., Rosenfeld, J.A., van Bon, B.W.M., Vulto-van Silfhout, A.T., Bosco, P., Friend, K.L., Baker, C., Buono, S., Vissers, L.E.L.M., et al. (2014). Refining analyses of copy number variation identifies specific genes associated with developmental delay. Nat. Genet. *46*, 1063–1071. https://doi.org/10.1038/ng.3092.
20. Kendall, K.M., Bracher-Smith, M., Fitzpatrick, H., Lynham, A., Rees, E., Escott-Price, V., Owen, M.J., O'Donovan, M.C., Walters, J.T.R., and Kirov, G. (2019). Cognitive performance and functional outcomes of carriers of pathogenic copy number variants: analysis of the UK Biobank. Br. J. Psychiatry *214*, 297–304. https://doi.org/10.1192/bjp.2018.301.
21. Crawford, K., Bracher-Smith, M., Owen, D., Kendall, K.M., Rees, E., Pardiñas, A.F., Einon, M., Escott-Price, V., Walters, J.T.R., O'Donovan, M.C., and Kirov, G. (2019). Medical consequences of pathogenic CNVs in adults: analysis of the UK Biobank. J. Med. Genet. *56*, 131–138. https://doi.org/10.1136/jmedgenet-2018-105477.
22. Gardner, E.J., Neville, M.D.C., Samocha, K.E., Barclay, K., Kolk, M., Niemi, M.E.K., Kirov, G., Martin, H.C., and Hurles, M.E. (2022). Reduced reproductive success is associated with selective constraint on human genes. Nature *603*, 858–863. https://doi.org/10.1038/s41586-022-04549-9.
23. Goodrich, J.K., Singer-Berk, M., Son, R., Sveden, A., Wood, J., England, E., Cole, J.B., Weisburd, B., Watts, N., Caulkins, L., et al.; AMP-T2D-GENES Consortia (2021). Determinants of penetrance and variable expressivity in monogenic metabolic conditions across 77,184 exomes. Nat. Commun. *12*, 3505. https://doi.org/10.1038/s41467-021-23556-4.
24. Tuke, M.A., Ruth, K.S., Wood, A.R., Beaumont, R.N., Tyrrell, J., Jones, S.E., Yaghootkar, H., Turner, C.L.S., Donohoe, M.E., Brooke, A.M., et al. (2019). Mosaic Turner syndrome shows reduced penetrance in an adult population study. Genet. Med. *21*, 877–886. https://doi.org/10.1038/s41436-018-0271-6.
25. Landrum, M.J., Lee, J.M., Benson, M., Brown, G., Chao, C., Chitipiralla, S., Gu, B., Hart, J., Hoffman, D., Hoover, J., et al. (2016). ClinVar: public archive of interpretations of clinically relevant variants. Nucleic Acids Res. *44*, D862–D868. https://doi.org/10.1093/nar/gkv1222.
26. Fry, A., Littlejohns, T.J., Sudlow, C., Doherty, N., Adamska, L., Sprosen, T., Collins, R., and Allen, N.E. (2017). Comparison of sociodemographic and health-related characteristics of UK biobank participants with those of the general population. Am. J. Epidemiol. *186*, 1026–1034. https://doi.org/10.1093/aje/kwx246.
27. Plomin, R., and Deary, I.J. (2015). Genetics and intelligence differences: five special findings. Mol. Psychiatr. *20*, 98–108. https://doi.org/10.1038/mp.2014.105.

The American Journal of Human Genetics, Volume *109*

Supplemental information

Rare genetic variants in genes and loci linked to dominant monogenic developmental disorders cause milder related phenotypes in the general population

Rebecca Kingdom, Marcus Tuke, Andrew Wood, Robin N. Beaumont, Timothy M. Frayling, Michael N. Weedon, and Caroline F. Wright

Supplementary Information

Supplementary Figures

Supplementary Figure 1: Histogram showing the Variant Allele Fraction (VAF) of variants located in *DNMT3A*, dashed line indicating the average VAF of variants in other genes, with filled line showing the average VAF of variants in *DNMT3A*.

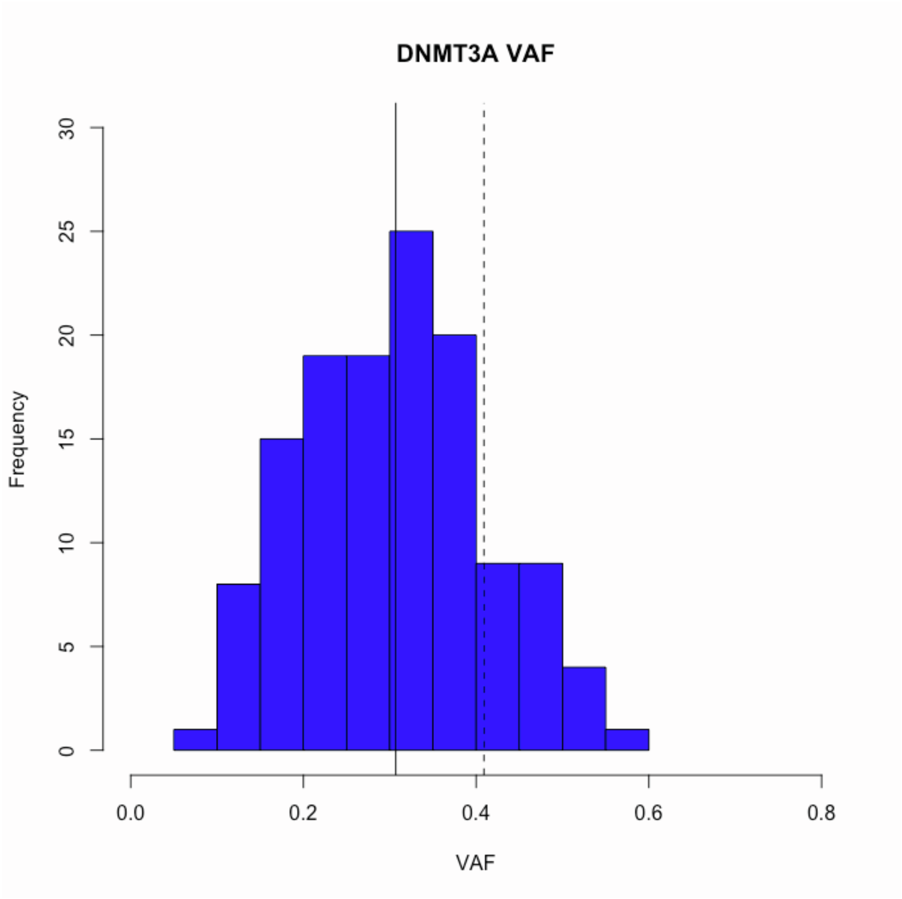

Supplementary Tables

Included in an Excel file.

Worksheet 1: Known monoallelic DDG2P genes

Worksheet 2: Copy number variants in UKB overlapping 69 known DD loci

Worksheet 3: Gene panel association tests for LoF variants across different gene subsets

Worksheet 4: Gene panel association tests for deleterious missense variants across different CADD bins

Worksheet 5: Gene panel association tests excluding individuals diagnosed with a childhood developmental disorder

Supplementary Methods

STATA code: Logistic regression

```
<import UKB BOLT phenotype file>
<import UKB exome file>
<import pcs file>
<import list of traits to test - varlist>

#varlist includes following traits: employed degree infertile epilepsy_ukbdef ChildDD_any
AdultDD_any unable_to_work

#for LoF variants:
generate variant = 1 if v2 == “lof_variant”
generate dont_use = 1 if v2 == “missense_variant”
replace variant = 0 if variant != 1
replace dont_use = 0 if dont_use != 1

file open myfile using <file_to_save_to>,
write append
set more off

foreach var of varlist {
	logistic `var' variant centre sex age_base pcs* if valid_exome =1 & dont_use != 1

file write myfile "`var'" _tab (r(table)[1,1]) _tab (r(table)[2,1]) _tab (r(table)[4,1]) _tab
(r(table)[5,1]) _tab (r(table)[6,1]) _n
}

file close myfile
```

STATA code: Linear regression

```
<import UKB BOLT phenotype file>
<import UKB exome file>
<import pcs file>
<import list of traits to test - varlist>

#varlist includes following traits: fluid_intelligence edu_yrs income reaction_time_raw_sin
pairs_test tdi age_education height mental_health numeric_memory_raw_sin
bmi_2016_raw_sin kids_fathered all_preg n_stillbirth

#for LoF variants:
generate variant = 1 if v2 == “lof_variant”
generate dont_use = 1 if v2 == “missense_variant”
replace variant = 0 if variant != 1
replace dont_use = 0 if dont_use != 1
```

```
file open myfile using <file_to_save_to>,
write append
set more off

foreach var of varlist {
	regress `var' variant centre age_base sex pcs* if valid_exome = 1 & dont_use != 1

file write myfile "`var'" _tab (r(table)[1,1]) _tab (r(table)[2,1]) _tab (r(table)[4,1]) _tab
(r(table)[5,1]) _tab (r(table)[6,1]) _n
}

file close myfile
```
